# Acute renal denervation normalizes aortic function and decreases blood pressure in spontaneously hypertensive rats

**DOI:** 10.1038/s41598-020-78674-8

**Published:** 2020-12-11

**Authors:** Nathalia Juocys Dias Moreira, Fernando dos Santos, Edson Dias Moreira, Daniela Farah, Leandro Eziquiel de Souza, Maikon Barbosa da Silva, Ivana Cinthya Moraes-Silva, Gisele Silvério Lincevicius, Elia Garcia Caldini, Maria Cláudia Costa Irigoyen

**Affiliations:** 1 Instituto do Coração da Faculdade de Medicina da Universidade de São Paulo (InCor-FMUSP), São Paulo, Brazil; 2 Escola Paulista de Medicina da Universidade Federal de São Paulo (EPM - UNIFESP), São Paulo, Brazil; 3 Departamento de Patologia, Faculdade de Medicina da Universidade de São Paulo (HC-FMUSP), São Paulo, Brazil

**Keywords:** Hypertension, Cardiovascular biology, Cardiology, Cardiovascular diseases

## Abstract

Mechanisms involved in the acute responses to renal denervation (RDN) have yet to be fully understood. We assessed urinary volume, autonomic control and aorta vascular reactivity after acute RDN. Male normotensive Wistar rats and spontaneously hypertensive rats (SHR) were divided into normotensive + RDN (ND) or sham surgery (NS), and hypertensive + RDN (HD) or sham surgery (HS). Metabolic parameters and hemodynamic measurements were recorded 72h and 4 days after intervention, respectively. Aortic rings were studied 7 days post RDN in an isometric myograph. Concentration–response curves to phenylephrine, sodium nitroprusside and acetylcholine (10^–10^–10^−5^ M) were performed. Two-way ANOVA was used for group comparisons and differences reported when p < 0.05. Results are presented as mean ± SEM. Urinary volume was 112% higher in HD *vs.* HS (HS = 14.94 ± 2.5 mL; HD = 31.69 ± 2.2 mL) and remained unchanged in normotensive rats. Systolic BP was lower in HD rats (HS = 201 ± 12 *vs*. HD = 172 ± 3 mmHg) without changes in normotensive group. HD group showed increased HF and LF modulation (HS = 5.8 ± 0.7 ms^2^ *vs*. HD = 13.4 ± 1.4 ms^2^; HS = 3.5 ± 0.7 ms^2^ *vs*. HD = 10.5 ± 1.7 ms^2^, respectively). RDN normalized vascular reactivity in HD rats and increased phenylephrine response in ND rats. Acute fall in BP induced by RDN is associated with increased urinary volume, which in turn may also have contributed to functional changes of the aorta.

## Introduction

It is known that amplitude of the degree of sympathetic nervous system activation is directly related to the magnitude of elevated blood pressure (BP) in the hypertensive patients^1^. In fact, in hypertension the sympathetic efferent fibers are hyperstimulated and contribute to increased BP^2^. The ablation of efferent renal nerves leads to a decrease in BP, since it reduces the activity of the renal sympathetic nerve, promoting urinary sodium excretion, whereas afferent renal sympathetic nerve ablation decreases BP by inhibiting central sympathetic flow^3^. Indeed, studies using experimental models have found that nerve ablation reduces both BP and the target organ damage caused by chronic sympathetic hyperactivity^4,5^. We have previously demonstrated the key role of renal nerves in cardiovascular homeostasis in hypertension induced by aortic ligation; complete denervation of the ischemic kidney was able to attenuate hypertension, normalize the renin plasma activity and baroreceptor reflex of heart rate 10 days after aortic ligation^6^.

Catheter-based renal denervation (RDN) has been proposed as a novel treatment in patients with resistant hypertension. Previous studies have shown the effectiveness of this procedure in reducing blood pressure in such patients^7,8^. Despite the enthusiasm surrounding the procedure, the Symplicity HNT-3 trial (2014), the first to compare renal nerve ablation with a sham procedure, found no significant reduction in the blood pressure. The failure of the trial has been attributed to several possible confounding factors, including issues related to patient selection and medication adherence, suboptimal procedural performance, and operator experience^9^. The more recent studies, with improved trial design, selection of relevant patient cohorts, and optimized interventional procedures have presented positive outcomes (change in 24-h blood pressure at 2, 3 and 6 months)^10–13^.

In light of the controversies still surrounding the effectiveness of this at present, RDN should for now be regarded as alternative approach, to be used only in patients within the context of clinical research and in highly qualified centers^14^.

Despite the accumulated knowledge gained over all these years of research, the mechanisms underlying BP reduction by RDN have yet to be fully understood. The beneficial effects of this technique on hypertension have been associated with mechanisms such as a progressive decrease in renin release and renal sodium retention^15^ and attenuation of oxidative stress and inflammation^16^.

Given the key role of the renal vasculature and the autonomic nervous system in conditions involving increased renal sympathetic nerve activity, such as hypertension and heart failure, the quest for a better understanding of these mechanisms seems to be crucial in proper inform the management and use of renal denervation procedure.

To our knowledge, there are no studies reporting changes in aortic vascular reactivity in renal denervated normotensive and spontaneously hypertensive rats (SHR). In order to have a fuller understanding of the mechanisms underlying RDN, we evaluated aorta response to phenylephrine (Phe), acetylcholine (Ach) and sodium nitroprusside (SNP). Our hypothesis is that renal nerve ablation may be able to improve aorta function. In addition, we assessed heart rate (HR) and blood pressure (BP) variability, along with the reflex control of HR as candidate mechanisms involved in the decrease in BP after denervation.

Therefore, the major aim of this study was to evaluate the autonomic modulation of the circulation of SHR animals after acute renal denervation (7–10 days), as well as the vascular function and morphological alterations of the thoracic descending aorta in these animals.

## Results

### RDN effects on blood pressure

**Figure 1:**
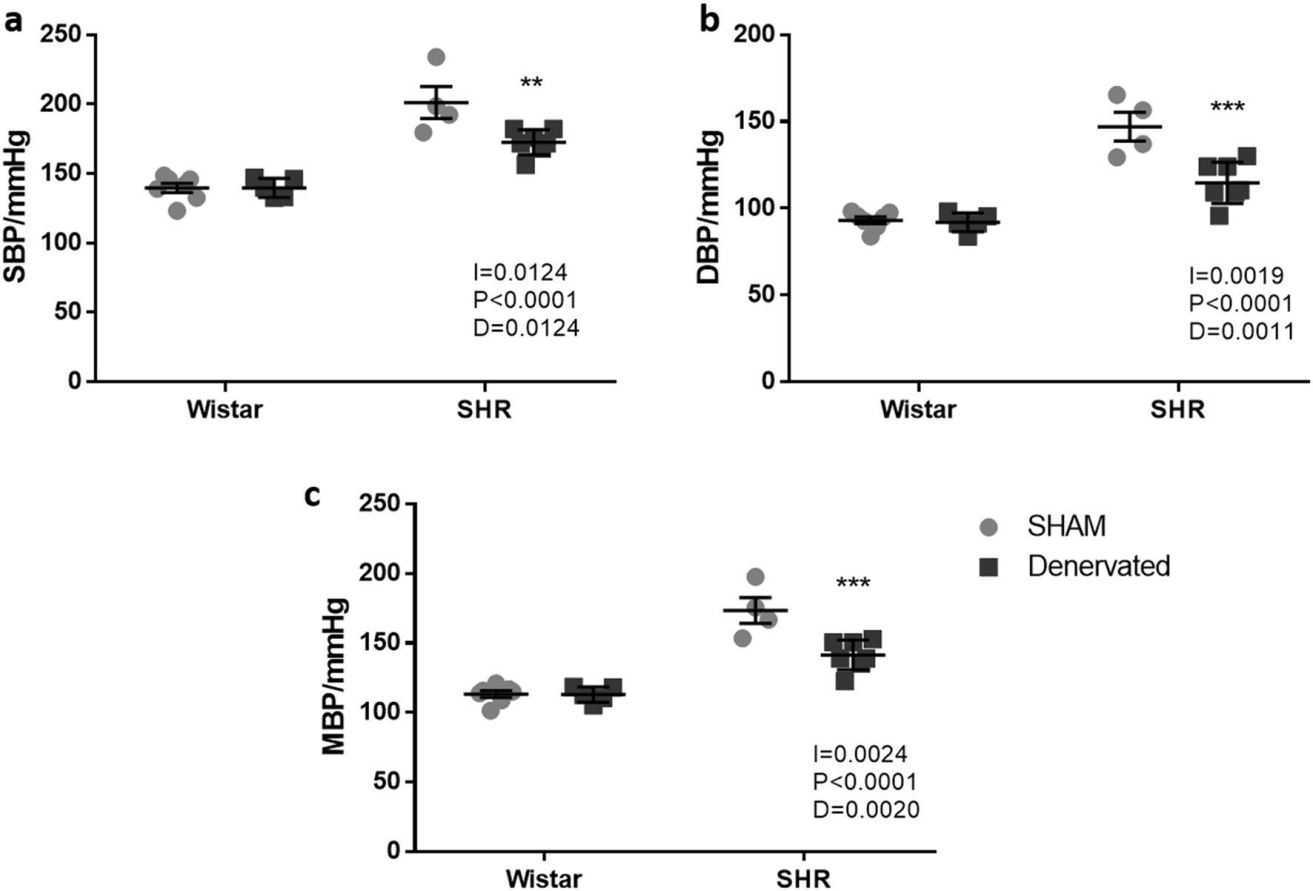
Blood pressure 4 days after intervention. (**a**) Systolic blood pressure (SBP); (**b**) diastolic blood pressure (DBP); (**c**) mean blood pressure (MBP). Data were analyzed by two-way ANOVA followed by Tukey’s post hoc test. Values are mean ± SEM. ANOVA: Interaction factor (I); Blood pressure background factor (P); Denervation factor (D). **p < 0.01; ***p < 0.001 *vs.* hypertensive sham (HS).

Although renal denervation was not able to normalize SHR rats blood pressure, hypertensive denervated rats showed lower systolic and diastolic pressures when compared to hypertensive sham group (HS = 201 ± 12 mmHg *vs.* HD = 172 ± 3 mmHg, and HS = 147 ± 8 mmHg *vs.* HD = 114 ± 4 mmHg, respectively), resulting in a lower mean pressure (HS = 175 ± 9 mmHg *vs.* HD = 141 ± 4 mmHg). However, normotensive group pressures remained unchanged, as showed in Fig. 1.

### RDN effects on autonomic balance

**Figure 2:**
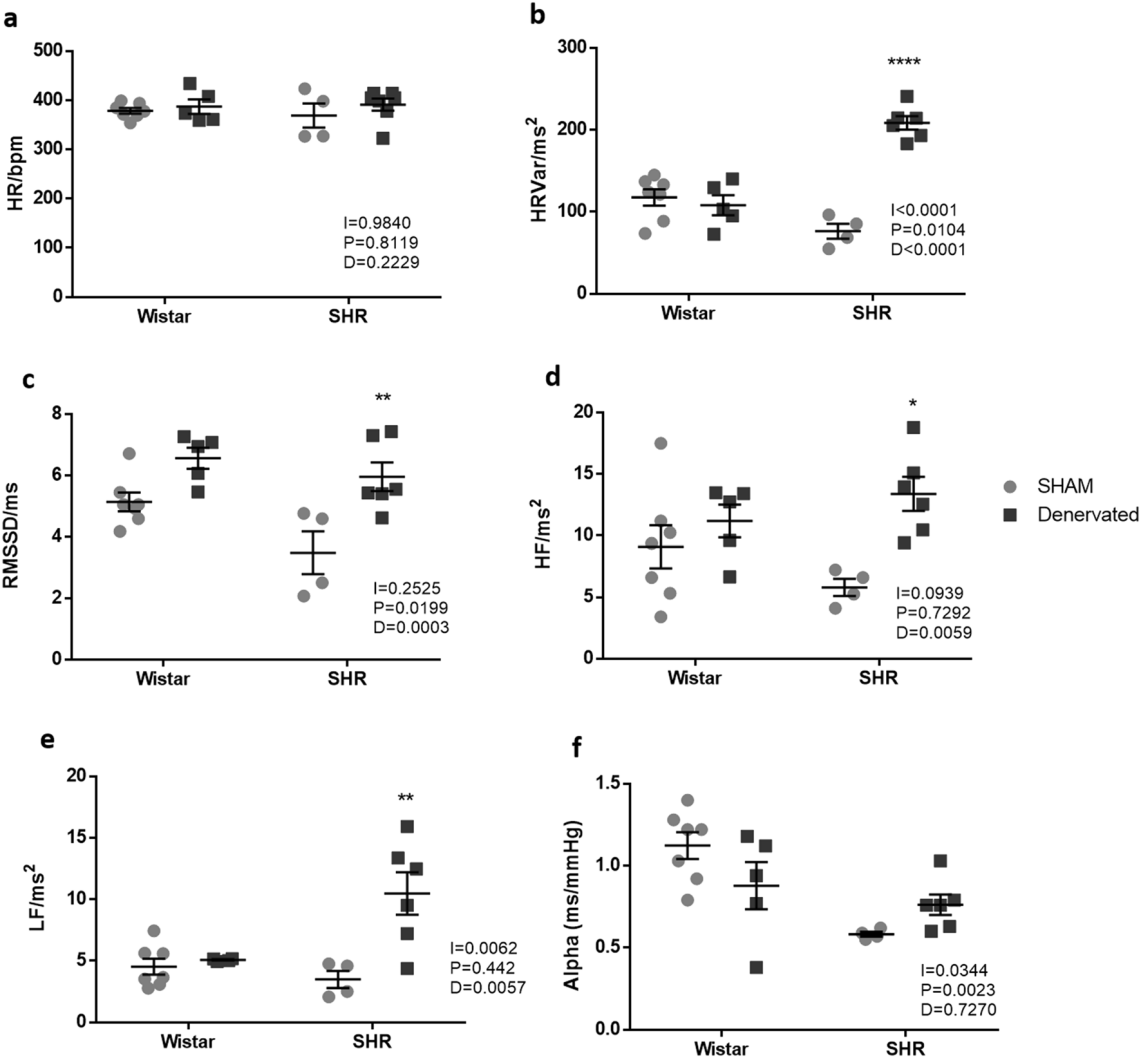
Autonomic balance 4 days after intervention. (**a**) Heart rate (HR); (**b**) heart rate variance (HRVar); (**c**) square root of the sum of the squares of the consecutive pulse interval differences (RMSSD); (**d**) high frequency (HF) modulation; (**e**) low frequency (LF) modulation. (**f**) Alpha index (AI). Data were analyzed by two-way ANOVA followed by Tukey’s post hoc test. Values are mean ± SEM. ANOVA: Interaction factor (I); Blood pressure background factor (P); Denervation factor (D). *p < 0.05; **p < 0.01; ****p < 0.0001 *vs*. hypertensive sham (HS).

Figure 2a to f shows RDN effects on autonomic balance. Although RDN did not change heart rate (HR) in normotensive Wistar or hypertensive rats, heart rate variance (HRVar) was higher in hypertensive rats when denervated (HS = 76 ± 9 ms^2^ *vs.* HD = 208 ± 8 ms^2^), while no difference was observed in Wistar rats (NS = 115 ± 10 ms^2^ *vs.* ND = 108 ± 12 ms^2^, Fig. 2a and b).

RMSSD and high frequency (HF) modulation, both involved in parasympathetic modulation, were greater in hypertensive denervated rats when compared to hypertensive sham (HS = 3.5 ± 0.7 ms *vs.* HD = 5.9 ± 0.5 ms; HS = 5.8 ± 0.7 ms^2^ *vs.* HD = 13.4 ± 1.4 ms^2^ respectively.

Should be emphasized that for these 2 parameters (RMSSD and HF) no differences between Wistar and RDN-treated SHR rats were found, indicating that renal denervation is capable to restore parasympathetic modulation in SHR. Normotensive rats remained unchanged (Fig. 2c and d)

Similarly, low frequency (LF), which reflects sympathetic modulation, was also increased in HD *vs.* HS (HS = 3.5 ± 0.7 ms^2^; HD = 10.5 ± 1.7 ms^2^) while no difference was observed for normotensive rats (NS = 4.5 ± 0.6 ms^2^; ND = 4.6 ± 0.5 ms^2^, Fig. 2e).

**Figure 3:**
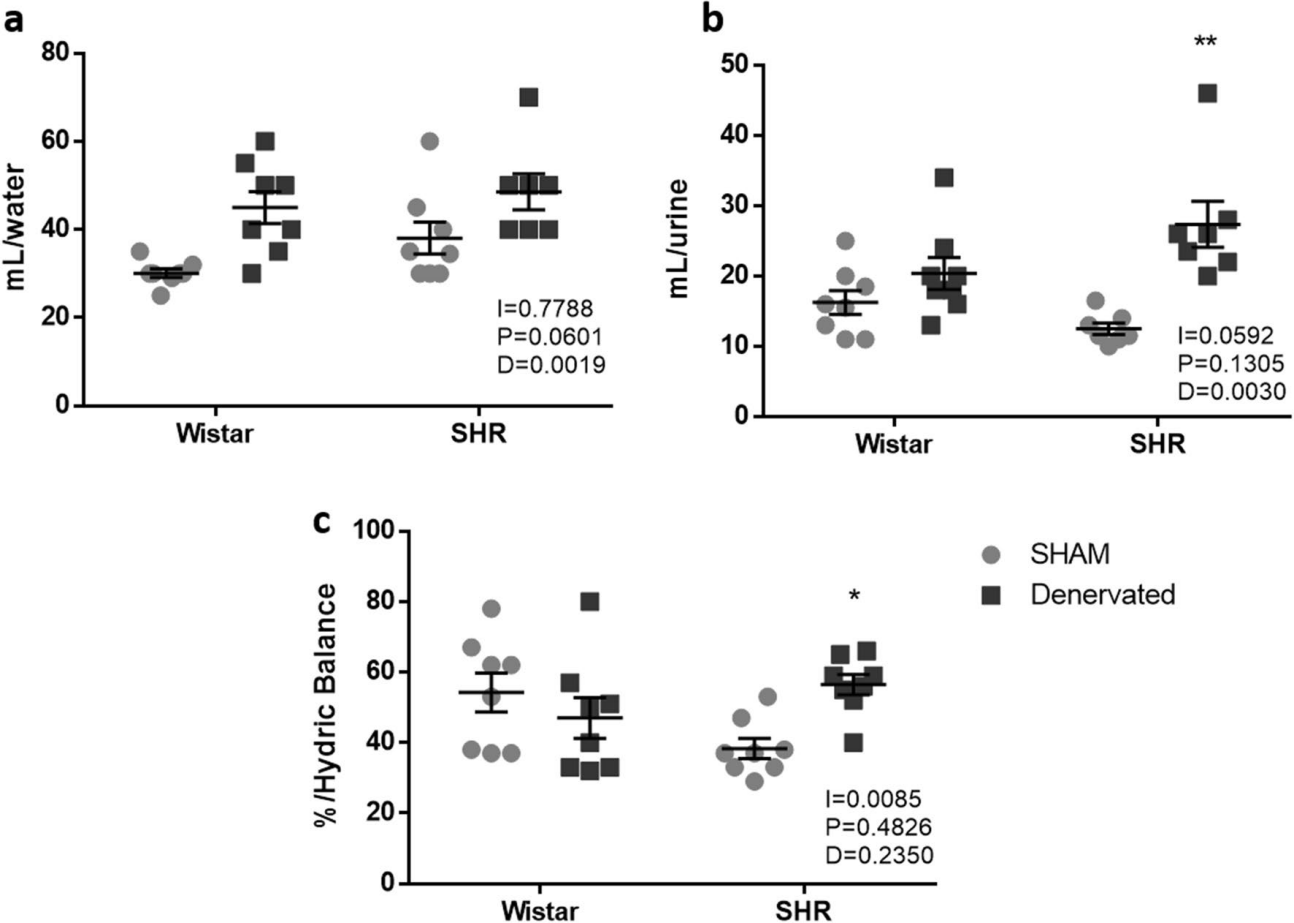
Metabolic parameters 72 h after intervention. (**a**) Water intake; (**b**) urinary volume; (**c**) hydric balance. Data were analyzed by two-way ANOVA followed by Tukey’s post hoc test. Values are mean ± SEM. ANOVA: interaction factor (I); blood pressure background factor (P); denervation factor (D). *p < 0.05; **p < 0.01 *vs*. hypertensive sham.

Alpha index (baroreflex marker) remained unchanged after RDN in both Wistar and SHR rats (Fig. 3f).

### RDN effects on metabolic parameters

Although hypertensive denervated animals (HD) ingested 46% more water than their control (HS), these same animals showed an urinary volume 112% higher than HS, ensuring greater hydric balance for this group (HS = 38 ± 3% *vs.* HD = 55 ± 3% ) and demonstrating volume loss in HD animals 72 h after intervention. However, no effects of RDN were observed on normotensive rats in urinary volume, water intake or hydric balance (Fig. 3). Body weight at the end of the experiment was NS = 399 ± 12 g; ND = 337 ± 11 g; HS = 294 ± 22 g and HD = 277 ± 4 g. Other metabolic parameters such as chow intake and feces are presented as supplementary data (see supplementary Fig. S1).

### RDN effects on aortic vasorelaxation response

**Figure 4:**
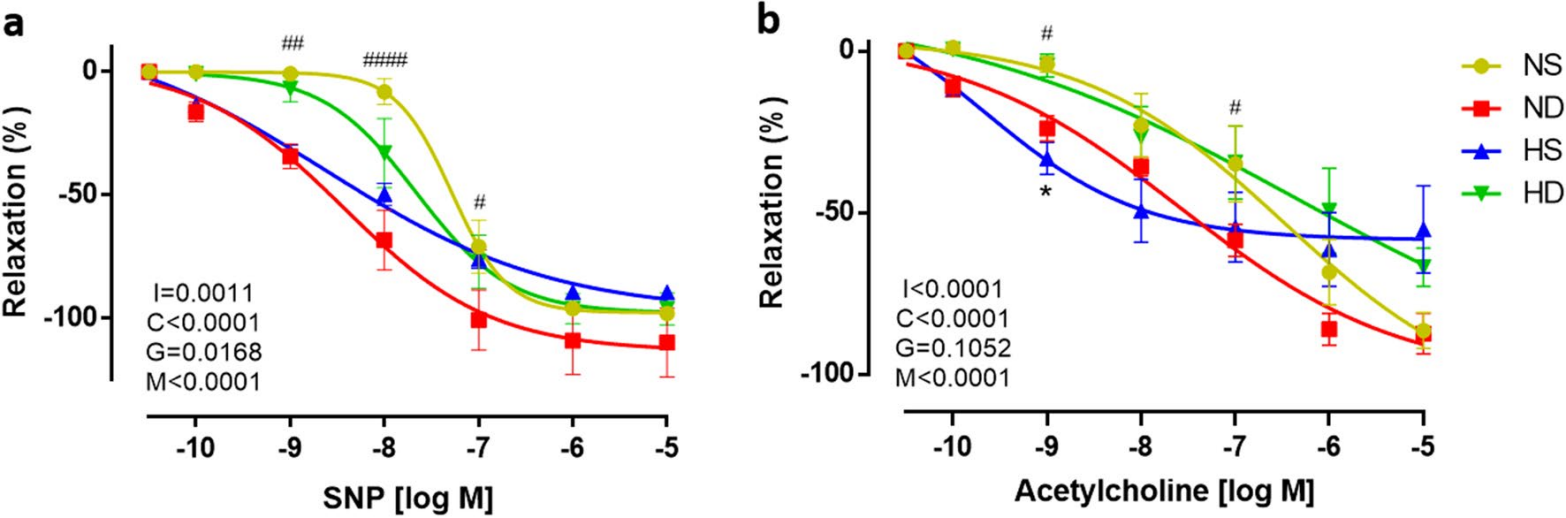
Aorta relaxation 7–10 days after renal denervation. (**a**) Concentration–response curve to sodium nitroprusside; (**b**) Concentration–response curve to Acetylcholine. NS: normotensive sham (n = 6); ND: denervated normotensive (n = 9), HS: SHR sham (n = 5); HD: denervated SHR (n = 4). Data were analyzed by two-way ANOVA followed by Fisher post hoc test. Values are mean ± SEM. ANOVA: Interaction factor (I); Concentration factor (C); Groups factor (G); Matching (M). #p < 0.05; ##p < 0.01; ####p < 0.0001 NS *vs.* ND; *p < 0.05 HS *vs.* HD.

Figure 4a and b represent aorta relaxation when vessel rings were exposed to a concentration–response curve to sodium nitroprusside (endothelium-independent relaxation), and acetylcholine (endothelium-dependent relaxation). When compared to normotensive sham rats (yellow circles), normotensive denervated rats (red squares) exhibited a left-down shift in vasodilator response. This is also observed in half-maximal dose (logED_50_) which shows a greater sensibility in ND to SNP (Table 1).

**Table 1:** Half-maximal dose (logED_50_) and maximal response (E_max_) to sodium nitroprusside (SNP), acetylcholine (Ach) and phenylephrine (Phe).

	NS	ND	HS	HD	p
**SNP**					
LogED_50_ (log M)	− 7.25 ± 0.16	− 7.88 ± 0.14^##^	− 7.75 ± 0.09	− 7.58 ± 0.18	I = 0.0202 P = 0.5303 D = 0.1585
E_max_ (%)	102.90 ± 0.80	109.90 ± 13.51	88.88 ± 1.68	97.77 ± 6.18	I = 0.9286 P = 0.2335 D = 0.4645
**Ach**					
LogED_50_ (log M)	− 6.98 ± 0.20	− 7.22 ± 0.07	− 8.37 ± 0.12	− 7.20 ± 0.11*	I = 0.0576 P = 0.0637 D = 0.2011
E_max_ (%)	86.55 ± 2.78	89.22 ± 5.50	57.58 ± 12.10	67.18 ± 6.06	I = 0.6590 P = 0.0035 D = 0.4370
**Phe**					
LogED_50_ (log M)	− 6.49 ± 0.20	− 6.71 ± 0.07	− 6.38 ± 0.12	− 6.28 ± 0.11	I = 0.2149 P = 0.0431 D = 0.6299
E_max_ (%)	1.00 ± 0.01	1.59 ± 0.21^#^	0.60 ± 0.06	1.19 ± 0.22	I = 0.9921 P = 0.0532 D = 0.0060

Values are mean ± SEM.

Data were analyzed by two-way ANOVA followed by Fisher post hoc test.

Normotensive sham (NS); denervated normotensive (ND); SHR sham (HS); denervated SHR (HS).

^#^p < 0.05, ##p < 0.01 *vs*. NS; *p < 0.05 *vs.* HS.

ANOVA: Interaction factor (I); blood pressure background factor (P); denervation factor (D).

On the other hand, although renal denervation did not change final relaxation to SNP in the hypertensive group, we observed a right up shift in HD group (green up-side down triangles) at lower concentrations when compared to HS (blue triangles). Also, in both SNP and Ach-induced relaxation the curve shifted to control values (green upside-down triangles *vs.* yellow circles). More importantly is the fact that hypertensive sham rats showed a contractile response in the three last doses of the Ach curve, indicating endothelial dysfunction, which did not occur in hypertension animals undergoing renal denervation. Endothelium-dependent and endothelium-independent relaxation in absolute values are presented as supplementary data (see supplementary Fig. S2).

### RDN effects on aortic contractile response

**Figure 5:**
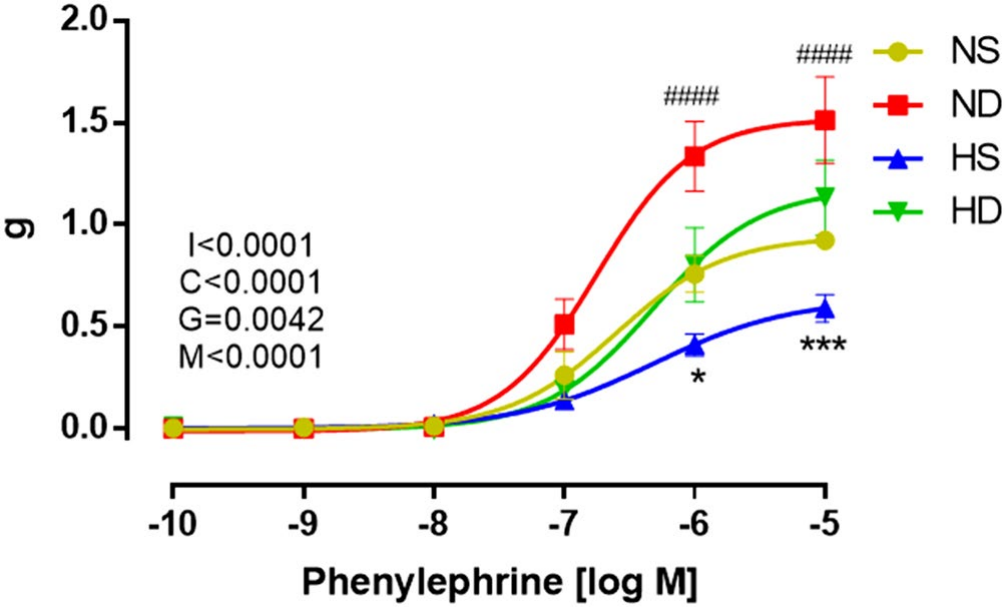
Concentration–response curve to phenylephrine. NS: normotensive sham (n = 6); ND: denervated normotensive (n = 9), HS: SHR sham (n = 5); HD: denervated SHR (n = 4). Data were analyzed by two-way ANOVA followed by Fisher post hoc test. Values are mean ± SEM. ANOVA: Interaction factor (I); Concentration factor (C); Groups factor (G); Matching (M). ####p < 0.0001 NS *vs*. ND; *p < 0.05; ***p < 0.001 HS *vs*. HD.

Figure 5 represents aortic contractile response when vessel rings were exposed to a concentration–response curve to phenylephrine (α1-agonist). Yellow circles represent Wistar sham group (control). When denervated (red squares), Wistar rats showed a greater contractile response when compared to control, also represented by a greater maximal response (E_max_, Table 1). The same behavior was observed in hypertensive animals; when denervated, SHR showed increased contractile response when compared to their control (blue triangles *vs.* green upside-down triangles). In summary, RDN increased the contractile response to phenylephrine for both normotensive and hypertensive rats, leading the latter to control values.

### RDN effects in aorta tunica media

**Figure 6:**
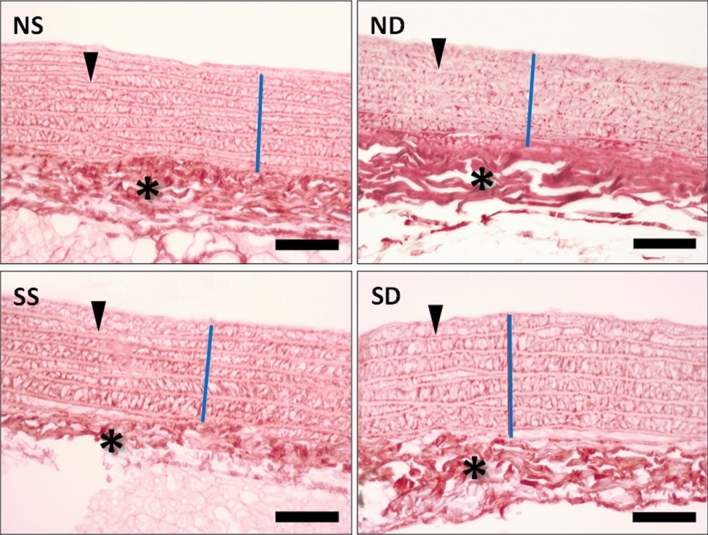
Representative images obtained from picrosirius-stained transverse aortic tissue sections from normotensive rats submitted to sham surgery (NS) or to renal denervation (ND), sham-operated spontaneous hypertensive rats (HS) or spontaneous hypertensive rats undergoing renal denervation (HD). Picrosirius staining demonstrates collagen in red and shows the typical histological organization of a normal aorta in all sections. The tunica media (blue lines) shows thin collagenous fibers interspersed among organized layers of smooth muscle cells and elastic lamellae (arrowheads). The tunica adventitia (asterisks) typically display coarse collagen fibers. Note that there is no difference between the groups for aorta morphology. Scale bars represent 50 µm.

As shown in Fig. 6, tissue sections showed characteristics of the healthy aortic tunicae (intima, media and adventitia). No histologic abnormalities were observed in the aortic wall, neither fibrosis, nor alteration of the arrangement of elastic lamellae, nor necrotic areas. Furthermore, there was no significant difference in the thickness of aortic tunica media among the experimental groups.

## Discussion

Our main findings regarding the effects of bilateral acute renal denervation in spontaneously hypertensive rats were: (1) The Blood pressure was significantly reduced 4 days after renal denervation; (2) Urinary volume was increased 72 h after renal denervation and (3) Aortic vascular function was improved by renal denervation.

Although it has been well documented that renal denervation—induced by different kinds of techniques (e.g. perivascular radiofrequency ablation, chemical ablation)—is able to lower blood pressure in SHR^17–22^, the effects of acutely renal denervation have not previously been fully addressed. While we believe that all measures should be carried out at the same times (simultaneously) of the experiment timeline, this was not always possible from a logistic perspective. However, evidences show that the effects of kidney denervation were extended at all times when such measures were taken^17,21^. In this way, renal denervation effectiveness has been evaluated by our group by both liquid chromatography technique (HPLC) or BP lowering effects as describe previously^23^.

Winternitz et al*.* have reported that 7 days after renal nerve ablation, SHR reaches a systolic blood pressure similar to normotensive animals^24^. Nevertheless, we found that 4 days after the procedure, renal denervation was already able to reduce SBP in SHR (14.4% of reduction) while BP in normotensive rats remain unchanged. On the other hand, a shorter period of renal denervation (1 h) did not change arterial pressure in SHR, despite the important changes observed in the intrarenal function in the same period—e.g., increase in renal blood flow (RBF), improvement in the dynamic autoregulation of RBF and increase in RBF variability^25^.

Regarding the effects of renal denervation on renal function, it is well documented that renal denervation produces an increase in sodium and water excretion and a decrease in the renal vascular resistance in some mammalian species^15,26–29^. Also, denervation in normal rats does not impact renal hemodynamics^30–32^. However, controversies surround the instant changes in renal hemodynamic and excretory functions following acute renal denervation. In the present study we found that the urinary volume was increased in the first 72 h after renal denervation in hypertensive rats, without changing urine flow in the normotensive animals. It is possible that the renal function of SHR was improved and the intrarenal balance was also modified by renal denervation as already suggested by Dibona and Sawin^25^. In this sense, we suggest that the acute reduction in the BP induced by renal denervation in the SHR is mediated—at least in part—by the increase in urinary volume. It has been proposed that a number of mechanisms—apart from renal function improvements—may underlie the beneficial effects of renal denervation on attenuation of hypertension. In this way, in our study SHR exhibited: Impaired baroreflex sensitivity^33,34^, increased renal sympathetic nerve activity^35,36^, decreased HR variance^37^ and autonomic imbalance^38,39^ which may also have been changed by renal denervation.

Heart rate variability (HRV) was the first non-invasive methodology extensively used to evaluate autonomic modulation of the sinus node in normal subjects and in patients with a range cardiac and non-cardiac diseases and to identify patients at increased risk for cardiac mortality^40^. Spectral analysis allows the identification and quantification of the major oscillations that characterize HRV particularly under resting controlled conditions. Interest in this approach has long been driven by the possibility of correlating short-term spectral components to neural discharge^41,42^ and of obtaining indirect information on neural modulation of the sinus node. Thus, HRV provides prognostic information to some diseases in which autonomic nervous system is involved^43^.

In the present study, RDN was able to increase both RMSSD and HF band—which are thought to be representative of cardiac parasympathetic modulation—in SHR rats; suggesting an increase in the vagal modulation induced by renal denervation. Interestingly, LF (sympathetic activity), was also increased in SHR, demonstrating an increase in total heart rate modulation, as confirmed by HRVar. We are aware of other possible mechanisms involved in LF band. One of the most probable confounding factors is the vascular volume change; it has been long acknowledge that volume withdrawal by hemodialysis, for example, may reduce LF modulation^44^. However, in our study, the HD group presented a higher volume excretion associated with higher LF levels, giving us more confidence about the sympathetic representativity role of LF band in this model. In addition, the RDN did not modify either HF or LF band in normotensive animals, suggesting that the removal of renal nerves is able to alter the autonomic control only in hypertensive animals presenting an impairment in the autonomic system.

Although SHR exhibited impaired baroreflex sensitivity and it has been suggested that it may be improved by autonomic modulation and a lower blood pressure^34,45^, we found no difference in alpha index, a marker of baroreflex sensitivity after renal denervation.

On the other hand, it has been shown that vascular dysfunction plays an important role in the SHR hypertension state. Endothelial dysfunction is characterized by decreased nitric oxide (NO) bioavailability and consequent impairment of endothelium-dependent relaxation to acetylcholine. Since this neurotransmitter needs an intact endothelium to induce relaxation, any endothelial dysfunction may impair this response^46–48^.

Spontaneously hypertensive rats presents key alterations in endothelial function^49,50^ along with impaired endothelium-dependent relaxation in aorta, femoral arteries and mesenteric resistance arteries^51–54^. Studies involving humans have found that impaired endothelial function may be strongly associated with increased cardiovascular risk and resistant hypertension^55^. Forearm endothelial dysfunction has been suggested as a marker of future cardiovascular events in patients with essential hypertension^56^. Furthermore, the severity of hypertension was positively associated with the degree of endothelial function impairment in The Framingham Heart Study^57^. Taking into account that high activity of sympathetic nerves may impair vascular endothelial function^58,59^, renal nerve removal may restore this function. Indeed, Yong Wang and colleagues, have demonstrated that RND may improve endothelial dysfunction in a rat model of type 2 diabetes mellitus with insulin resistance^60^. In the current study, SHR presented an impaired endothelium-dependent relaxation as well as a sustained vasoconstrictor response on the three last Ach concentrations. However, RDN-treated hypertensive rats exhibited proper endothelium-dependent relaxation (not different from the control group, NS), suggesting that renal denervation was able to improve endothelial function in SHR rats 7–10 days after the intervention. Although a left down shift was observed in normotensive RDN-treated rats, no endothelial dysfunction was found, since curve behavior was similar to control, with corresponding final relaxation and no vasoconstriction at any concentration.

Because endothelial function is also highly dependent on nitric oxide (NO) bioavailability^61^, we challenged aortic rings to a concentration–response curve to SNP (nitric oxide donor that leads to an endothelium-independent relaxation), whose effect is attributed to its direct action on the vascular smooth muscle^62^. SNP concentration–response curve demonstrated that RDN abolished the difference between hypertensive and normotensive rats, suggesting that renal nerve removal improved NO bioavailability in hypertensive individuals. These findings corroborates a study carried out by Patel et al., who found that renal denervation was able to restore neuronal NO synthase (nNos) down regulation within paraventricular nucleus in rats with heart failure^63^. They also corroborate an investigation undertaken by Yong Wang et al., who reported significantly NO level increases after RDN in a rat model of type 2 diabetes mellitus with insulin resistance^60^. All the conditions mentioned above (hypertension, heart failure, type 2 diabetes) are often associated with sympathetic nervous system hyperactivity^64–68^. In this context, is likely that in the currently study, NO bioavailability is at some level being modulated by sympathetic nervous system. In normotensive denervated rats, not only a left-down shift in the curve was seen, but also a greater sensitivity to SNP, assessed by half-maximal response—logED_50_ (Table1). Nevertheless, the current study cannot discriminate whether logED_50_ value is due a greater sensitivity to SNP or due an increase in NO bioavailability. Interestingly, although NO bioavailability may be increased in RDN-treated rats, phenylephrine (α1-receptor agonist) evoked greater contractile response in both Wistar and SHR. It is possible that this increase may be associated with a previously described contractile potential of NO^69^.

SHR rats have reduced aorta responses to different vasoconstrictors such as norepinephrine^77^, phenylephrine, angiotensin II, endothelin-1 and urotensin-II^70^. In the present study, we demonstrated contractile activity induced by phenylephrine, and we found a lower response in hypertensive sham animals compared to Wistar rats. In the other hand, we observed a higher contractility induced by renal denervation in both hypertensive and normotensive animals. Studies have suggested that renal denervation may lead to an augmentation in the expression of α1-adrenoceptors, which in turn increases the response to neural and adrenergic stimuli^71,72^. The augmented contractile response to phenylephrine in hypertensive RDN-treated rats could be due to the increased sensitivity of these adrenergic receptors against the reduction of peripheral sympathetic activity promoted by renal denervation^73^. This response is likely to be associated with a different baseline tone between normotensive and hypertensive rats.

Although it has been suggested that aorta from SHR undergo early remodeling that leads to reduced contractility in vitro^70^, our data do not support this hypothesis, since we observed no difference in aorta tunica media layer thickness among groups.

Hypertensive individuals present vascular fibrosis and increased intima-media relationship, leading to an increased risk of cardiovascular events^74^. Thus, the degree of artery remodeling has a prognostic value in these individuals. Santos et al*.* have found outward hypertrophic remodeling and increased elastic fibers content on aortas of 9 month SHR rats^75^. Honl et al*.* have shown that renal denervation prevented reduction of aortic distensibility in atherosclerosis prone ApoE‑deficient rats 8 weeks after intervention^76^. In humans, data from a multi-center trial showed that aortic distensibility is improved 6 months after RDN regardless of the BP response to the intervention^77^. It should be emphasized that our animal vessels were evaluated 7 days after RDN. As we assessed acute response to renal denervation, a higher degree of remodeling would probably be found in older animals for chronic response.

## Conclusion

Spontaneously hypertensive rats had impaired endothelial function, as demonstrated by SNP, Ach and Phe concentration–response curves. Renal denervation normalized either dependent and independent—endothelium vasorelaxation in SHR and improved Phe contraction in both SHR and normotensive rats. The changes found in aorta responses may shed new light on the effects of renal denervation. We suggest that α1-adrenoreceptors and nitric oxide bioavailability may be the key mechanisms involved in this process and they possibly play a critical role in vascular changes after renal nerve removal, but new assessments may clarify this hypothesis.

Finally, acute responses to RDN indicate that the fall in BP is probably associated with the increased urinary volume, which may also have contributed to aortic functional changes. Baroreflex set point shifting may have participating to new BP values adjustments.

## Materials and methods

### Bioethical statement

We declare that all methods used in this protocol were carried out in accordance with relevant guidelines and regulations. All experiments were carried out after approved by the Ethical Committee for Animal Use from Faculdade de Medicina da Universidade de São Paulo (FMUSP) and the Research Ethics Committee of the Universidade Federal de São Paulo (UNIFESP) under protocol number 125/14 and 9324210514 respectively.

### Experimental protocol

**Figure 7:**
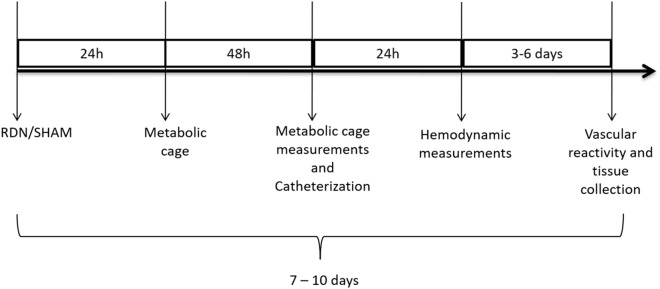
Schematic timeline protocol. RDN = renal denervation; h = hours.

To evaluate the autonomic modulation of the circulation after renal nerves ablation, male Wistar and SHR rats were divided in four experimental groups; NS (normotensive + sham surgery), ND (normotensive + RDN surgery), HS (hypertensive + sham surgery), HD (hypertensive + RDN surgery). Metabolic parameters and blood pressure were evaluated 72 h and 4 days after surgery respectively. Blood pressure records were used for hemodynamic measurements and autonomic evaluation, as described below. At the end of the protocol the animals were euthanized through anesthetic overdose and the aorta function assessed using a wire myograph (DMT 620 M). Aortas were collected for histological analysis. The timeline and schedule of the protocol is illustrated in Fig. 7.

### Animals

Male Wistar and spontaneously hypertensive rats (300–450 g, aged 12 weeks) were obtained from the Center for the Development of Experimental Models for Biology and Medicine (CEDEME). The animals were kept in individual cages, housed in the Laboratório de Hipertensão Experimental of Instituto do Coração/FMUSP, at room temperature (22–24 °C) and under controlled lighting, in a light/dark cycle (12 h). They were given access to rat chow and water ad libitum. The animals were randomly assigned into 4 (four) experimental groups: Wistar normotensive rats undergoing sham surgery (NS, n = 7); Wistar normotensive rats undergoing bilateral renal denervation (ND, n = 10); spontaneously hypertensive rats undergoing sham surgery (HS, n = 6), and spontaneously hypertensive rats undergoing bilateral renal denervation (HD, n = 8).

### Renal denervation (RDN)

The RDN surgical procedure was adapted and validated by Oliveira et al.^6^. The animals were fasted for eight hours before the procedure and then anesthetized with isoflurane 2.5% in a 0.8 L/min of O_2_ flow. A trichotomy was performed on the right flank along with the cleansing of the site with 70% ethyl alcohol. The method consisted in placing the animal in the lateral decubitus position and after an incision in the left flank and identification of the kidney, it was folded in such a way as to expose the region of the aorta and renal vein. The section of renal nerves was then removed from the adventitia around the vessels using an iris clamp and ophthalmic scissors, with subsequent general cleansing of the extension—from the abdominal aorta to the kidney—in order to avoid any remaining nerve fibers. The same procedure was performed on the left side. The entire procedure was carried out with the use of magnifying glass and field magnification of 16× (M900, DF Vasconcellos).

The animals undergoing sham surgery had the renal artery and vein region exposed, followed by mild manipulation with a cotton tip. The incision suture was performed in two stages: suture of the muscular layer and skin suture, both using 4.0 nylon thread.

### Metabolic parameters

Twenty-four hours after renal denervation, the animals were placed in individual metabolic cages for measuring metabolic parameters, such as water, urinary excretion, chow intake and feces. During the first 24 h, the animals were adapted to the housing conditions, and the data were collected over the previous 24 h.

### Catheterization

When removed from metabolic cages, animals were anesthetized with 2.5% isoflurane in a 0.8 L/min of O_2_ flow and femoral artery and vein were catheterized as previously described^78^ to allow blood pressure recording and drugs infusion respectively.

### Hemodynamic assessments

#### Heart rate and blood pressure evaluation

BP measurement was performed 24 h after the catheterization procedure (4 days after RDN).

During blood pressure recording, the animals remained awake and allowed to move freely in the cage. BP signals were recorded over a period of 30 min on a microcomputer equipped with a data acquisition system (Windaq, 2KHz, DATAQ Instruments, Akron, OH, USA) to obtain pressure pulses, beat-to-beat (with a sampling frequency of 4000 Hz per channel) for the assessment of systolic blood pressure (SBP), diastolic blood pressure (DBP), mean blood pressure (MBP) and heart rate (HR). HR values were derived from the pulsatile BP signal.

### Autonomic balance assessment

#### Heart rate, blood pressure variability and baroreflex sensitivity

Systolic blood pressure (SBP) and Heart Rate (HR) variability were analyzed in time domain, which involved calculating the mean values of the pulse interval and SBP. Variability of these same variables was quantified by standard deviation, variances and the square root of the sum of the squares of the consecutive pulse interval differences (RMSSD). For analysis in frequency domain, a spectral analysis of the basal registers was performed using the Fast Fourier Transform (FFT) method. Power was obtained using time series derived from pulse interval and blood pressure, with a 512-point Hanning window and 50% overlap (CardioSeries v2.4). The powers for the low frequency bands (LF, 0.20–0.75 Hz, sympathetic modulation) and high frequency (HF, 0.75–3.0 Hz, parasympathetic modulation) were calculated by integrating the power in the bands of interest and presented as absolute values, percentages and were then normalized. For normalization, the potencies of the LF and HF bands were divided by the subtracted power variance in the very low frequency band^79^. The coupling between pulse interval and systolic blood pressure was estimated by the coherence function. A coherence value (K) greater than 0.5 was considered significant.

Baroreflex sensitivity was measured through alpha index of LF band, calculated by the square root of the ratio between the absolute value of LF PI by the absolute value of LF SBP, correlating the absolute values of LF for the heart with absolute LF for the vessels, when they were consistent with each other^80^.

### Functional evaluation of the descending aorta

In order to evaluate the functional unit of the aorta, vascular reactivity was performed using a wire myograph (DMT 620 M). For this, the animals were euthanized with anesthetic overload (thiopental sodium 80 mg/kg) 7–10 days after RDN or sham surgeries. The thoracic aorta was removed, dissected from the connective tissue and cut into approximately 2 mm length rings, which were carefully transferred to organ baths in the myograph to preserve their endothelial and elastic integrity.

Conductance arteries with an inner diameter of approximately 1200 μm remained one hour at baseline in 5 ml of Krebs solution containing 118.6 mmol L^−1^ NaCl; 4.7 mmol L^−1^ KCl; 2.5 mmol L^−1^ CaCl_2;_ 1.2 mmol L^−1^ MgSO_4;_ 1.2 mmol L^−1^ KH_2_PO_4;_ 25.1 mmol L^−1^ NaHCO_3_ and 11.1 mmol L^−1^ glicose; at 37º C, pH = 7.4 and under constant aeration with carbogenic mixture (95% O2 and 5% CO2). After stabilization period, aortic rings underwent a concentration–response curve to phenylephrine (Phe; 10^–10^ a 10^−5^ M), administered cumulatively in the bath to evaluate vasoconstrictor function and then washed with Krebs solution and left for 1 h for a new basal period. Subsequently, acetylcholine (Ach; 10^–10^ to 10^−5^ M) concentration–response curve was performed to evaluate endothelium-dependent vasodilation capacity and assess its integrity; vessels were previously contracted with 1 μM/L phenylephrine. Concentration–response curve was also performed on sodium nitroprusside to assess the endothelium-independent vasodilatory capacity; sodium nitroprusside (SNP; 10^–10^ a 10^-5^ M) was added to the bath solution on vessels previously contracted with 1 μM/L phenylephrine. All data were recorded by the Powerlab acquisition system and Chart 4.0 software (AD Instruments).

Half-maximal dose (logED_50_) and maximal response (E_max_) to sodium nitroprusside, acetylcholine and phenylephrine were calculated as previously described^81,82^. The log dose that produced a 50% of maximal response (ED_50_) reflects sensitivity to the drugs; lower ED_50_ reflects higher sensitivity, whereas a higher ED_50_ reflects lower sensitivity.

### Perfusion and tissues collection

Animals which were not included in vascular reactivity analysis were euthanized (7–10 days after RDN or sham surgeries) by anesthetic overload (thiopental sodium 80 mg/kg) followed by perfusion with physiological solution and Potassium Chloride (14 mmol/L). The tissues were then perfused with formaldehyde (10%) and the thoracic aorta collected for subsequent histological analysis.

### Aorta medial layer thickness

Aorta tissues were stained in Picrossirius red, wich consisted of cutting the tissue using a microtome and then subjecting it to dehydration, whitening and inclusion it in paraffin. The cuts obtained were kept in an oven at 37ºC for 24 h and then the following steps took place: (1) Dewaxing in Xylol baths; (2) Hydration in decreasing alcohol baths (100–50%, followed by distilled water bath); (3) Immersion of tissues in Picrossirius red solution for one hour; (4) Washing under running water to remove excess dye; (5) Dehydration in increasing alcohol baths (50–100%); (6) Blade assembly.

Once the slides were assembled, they were placed under a microscope (Carl Zeiss AxioImager A.1) and observed at 200× magnification. The middle layer thickness of the descending aortas was evaluated by measuring the intima layer until the intersection of the middle layer with the adventitial layer (Quantimed). This measurement was performed at three different points in the vessel and was expressed as the mean of these values in micrometers (μm).

### Statistical analysis

The results are presented as mean ± SEM. Two-way ANOVA was used to compare both denervation and blood pressure background factors, followed by Tukey’s post hoc test. For vascular reactivity, two-way ANOVA for repeated measures was performed followed by Fisher post hoc test. Values p < 0.05 were considered statistically significant.

## Supplementary Information

Supplementary Information.
