# Supplementary material for: Acute renal denervation normalizes aortic function and decreases blood pressure in spontaneously hypertensive rats
Source: Sci Rep. 2020 Dec 11;10:21826. doi: 10.1038/s41598-020-78674-8 (PMC7733454; doi:10.1038/s41598-020-78674-8)
Supplement: Supplementary file 1 — Supplementary Information. [file 41598_2020_78674_MOESM1_ESM.pdf]

ACUTE RENAL DENERVATION NORMALIZES AORTIC FUNCTION AND DECREASES BLOOD PRESSURE IN SPONTANEOUSLY HYPERTENSIVE RATS

Nathalia Juocys Dias Moreira[1,2]; Fernando dos Santos[1]; Edson Dias Moreira[1]; Daniela Farah[1,2]; Leandro Eziquiel de Souza[1]; Maikon Barbosa da Silva[1]; Ivana Cinthya Moraes - Silva[1]; Gisele Silvério Lincevicius[1,2]; Elia Garcia Caldini[3]; Maria Cláudia Costa Irigoyen[1*].
[1]Instituto do Coração da Faculdade de Medicina da Universidade de São Paulo, São Paulo (InCor-FMUSP); [2]Escola Paulista de Medicina da Universidade Federal de São Paulo, São Paulo (EPM - UNIFESP); [3]Departamento de Patologia da Faculdade de Medicina da Universidade de São Paulo, São Paulo (HC-FMUSP). E-mail: hipirigoyen@incor.usp.br

Figure S1 A and B represents food intake (chow) and feces during metabolic cage period (24h post renal denervation). Hypertensive denervated rats ingested less chow and as consequence excreted less feces when compared to hypertensive sham group. Normotensive animals remained unchanged.

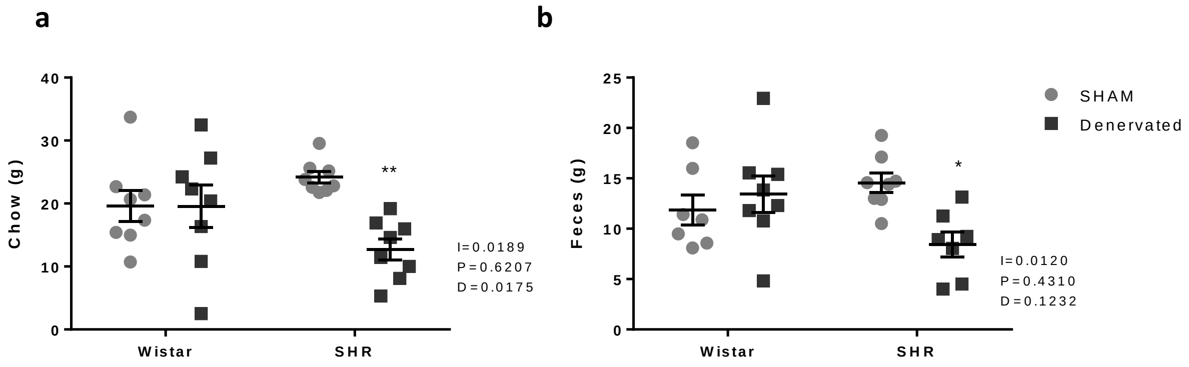

Figure S1: Metabolic parameters 24 to 48h after intervention. A) Chow intake; B) Feces. Data were analyzed by two-way ANOVA followed by Tukey’s post hoc test. Values are mean±SEM. ANOVA: Interaction factor (I); Blood pressure background factor (P); Denervation factor (D). *p<0.05; **p<0.01 vs. HS.

Absolute values of endothelium-independent (sodium nitroprusside) and endothelium-dependent (acetylcholine) relaxation are represented in figure S2 A and B, respectively. Hypertensive sham rats showed an impaired relaxation while hypertensive rats that undergo renal denervation normalized aorta relaxation.

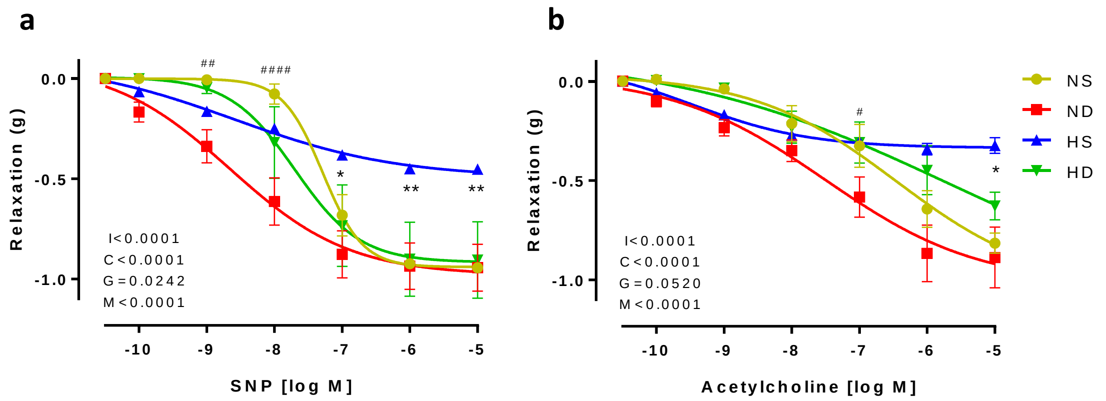

Figure S2: Aorta relaxation 7-10 days after renal denervation, presented as absolute values. A) Concentration-response curve to sodium nitroprusside. B) Concentration-response curve to Acetylcholine. NS: normotensive sham (n=6); ND: denervated normotensive (n=9), HS: SHR sham (n=5); HD: denervated SHR (n=4). Data were analyzed by two-way ANOVA followed by Fisher post hoc test. Values are mean±SEM. ANOVA: Interaction factor (I); Concentration factor (C); Groups factor (G); Matching (M). #p<0.05; ##p<0.01; ####p<0.0001 NS vs. ND. *p<0.05; **p<0.01 HS vs. HD.
